# Enzymatic Strategies to Detoxify Gluten: Implications for Celiac Disease

**DOI:** 10.4061/2010/174354

**Published:** 2010-10-07

**Authors:** Ivana Caputo, Marilena Lepretti, Stefania Martucciello, Carla Esposito

**Affiliations:** ^1^Department of Chemistry, University of Salerno, 84084 Salerno, Italy; ^2^European Laboratory for the Investigation of Food-Induced Diseases, University Federico II, 80131 Naples, Italy

## Abstract

Celiac disease is a permanent intolerance to the gliadin fraction of wheat gluten and to similar barley and rye proteins that occurs in genetically susceptible subjects. After ingestion, degraded gluten proteins reach the small intestine and trigger an inappropriate T cell-mediated immune response, which can result in intestinal mucosal inflammation and extraintestinal manifestations. To date, no pharmacological treatment is available to gluten-intolerant patients, and a strict, life-long gluten-free diet is the only safe and efficient treatment available. Inevitably, this may produce considerable psychological, emotional, and economic stress. Therefore, the scientific community is very interested in establishing alternative or adjunctive treatments. Attractive and novel forms of therapy include strategies to eliminate detrimental gluten peptides from the celiac diet so that the immunogenic effect of the gluten epitopes can be neutralized, as well as strategies to block the gluten-induced inflammatory response. In the present paper, we review recent developments in the use of enzymes as additives or as processing aids in the food biotechnology industry to detoxify gluten.

## 1. Celiac Disease

Celiac Disease (CD) is a condition affecting 1:70–1:200 individuals worldwide that may be diagnosed at any age [1, 2]. In a population-based study, increasing prevalence and high incidence of CD (1:47) in elderly people (older than 52 years of age) have been remarked [3]. CD is a permanent food intolerance to the ingested gliadin fraction of wheat gluten and similar alcohol-soluble proteins of barley and rye in genetically susceptible subjects [4, 5]. Most patients tolerate oats without any signs of intestinal inflammation probably because oat avenins are phylogenetically more distant from the analogous proteins in wheat, rye, and barley [6]. Nonetheless, a few individuals with clinical oat intolerance have avenin-reactive mucosal T cells that can cause mucosal inflammation [7]. In children prone to CD, exposure to wheat, barley, and rye in the first three months of life significantly increases the risk of developing CD compared with exposure between 4 and 6 months [8] whereas breastfeeding exerts a protective effect and the risk of CD is reduced if children are still being breast-fed when dietary gluten is introduced [9].

The clinical features of CD vary considerably [2]. Intestinal symptoms are frequent in children diagnosed within the first two years of life. However, asymptomatic patients can be found: failure to thrive, chronic diarrhoea, vomiting, abdominal distension, muscle wasting, anorexia, and general irritability are present in most cases. The wider use of serological screening tests is making it easier to recognize extra-intestinal manifestations such as short stature, anaemia unresponsive to iron therapy, osteoporosis, ataxia, peripheral neuropathies, hypertransaminasemia, and unexplained infertility [10]. It is also noteworthy that CD is associated with a high prevalence of concomitant autoimmune diseases (approximately 5–10 times greater than in the general population), such as endocrine autoimmune diseases, thyroid diseases, and selective IgA deficiency, as well as of genetic disorders, such as Down and Turner's syndromes [11]. 

Because symptoms may improve with a gluten-free diet, it is thought that gluten plays a key role in the pathogenesis of this disease.

## 2. Gluten

The grain protein content of wheat varies between 8 and 17 percent, depending on genetic make-up and external factors associated with the crop. A unique property of wheat flour is that, when in contact with water, the insoluble protein fraction forms a viscoelastic protein mass known as gluten. Gluten, which comprises roughly 78 to 85 percent of the total wheat endosperm protein, is a very large complex mainly composed of polymeric (multiple polypeptide chains linked by disulphide (SS) bonds) and monomeric (single chain polypeptides) proteins known as glutenins and gliadins, respectively. Gliadin consists of proteins containing *α*/*β*-, *γ*-, and *ω*-gliadins. In contrast to *α*/*β*- and *γ*-gliadins, which form three and four intramolecular (SS) bonds, respectively, *ω*-gliadins lack cysteine residues. Glutenin is a heterogeneous mixture of SS-linked polymers with a largely unknown polymer structure. A glutenin polymer consists of glutenin subunits of high or low molecular weight that are connected by intermolecular SS bonds. Glutenins confer elasticity, while gliadins mainly confer viscous flow and extensibility to the gluten complex. Thus, gluten is responsible for most of the viscoelastic properties of wheat flour doughs, and it is the main factor dictating the use of a wheat variety in bread and pasta making. Gluten viscoelasticity, for end-use purposes, is commonly known as flour or dough strength [12, 13].

Gliadins and glutenins have a unique amino acid composition with a high content of proline (15%), hydrophobic amino acids (19%), and glutamine (35%), hence they are named prolamins. Moreover, they contain domains with numerous repetitive sequences rich in those amino acids. Because of this glutamine- and proline-rich structure, gluten proteins are resistant to complete digestion by pancreatic and brush border proteases [14, 15].

## 3. Posttranslational Modification of Gluten by Tissue Transglutaminase

CD is triggered by an inappropriate T cell-mediated immune response to dietary gluten proteins. Consequently, CD patients display various degrees of intestinal inflammation, ranging from mild intraepithelial lymphocytosis to severe subepithelial mononuclear cell infiltration that results in total villous atrophy coupled with crypt hyperplasia. The most evident expression of autoimmunity in CD is the presence of serum antibodies to tissue transglutaminase (tTG), the main autoantigen of endomysial antibodies [16]. 

tTG is a member of a Ca^2+^-dependent enzyme family involved in post-translational modifications of proteins. tTG prevalently catalyzes the formation of stable isopeptide bonds between the *γ*-carboxamide group of the protein-bound glutamine residue and an appropriate amino group, either the *ε*-amino group of a protein-bound lysine residue or a small biogenic amine molecule such as putrescine, spermine, spermidine, and histamine. However, the absence of suitable nucleophilic amines and a low pH favours tTG deamidation of protein-bound glutamine residues [17]. 

**Figure 1:**
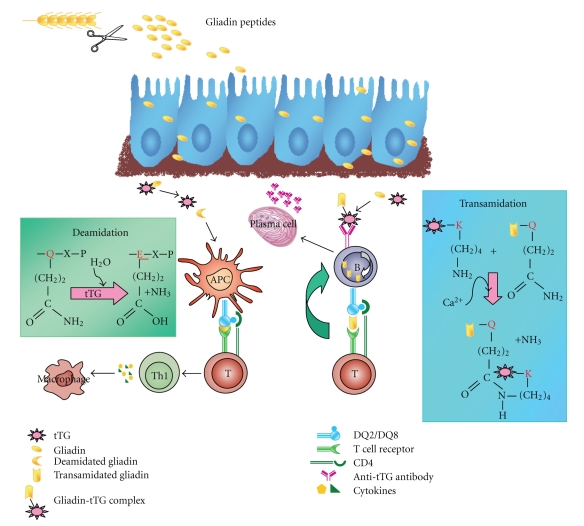
Tissue transglutaminase (tTG)-mediated post-translational modifications in celiac disease. Gliadin peptides reach the subepithelial region of the intestinal mucosa. Here, tTG deamidation of specific glutamines of gliadin peptides generates potent immunostimulatory epitopes that are presented via HLA-DQ2/DQ8 on antigen-presenting cells (APC) to CD4^+^ T cells. Activated gliadin-specific CD4^+^ T cells produce high levels of pro-inflammatory cytokines, thus inducing a Th_1_ response that results in mucosal remodelling and villous atrophy. In addition, tTG transamidation activity generates tTG-gliadin complexes that bind to tTG-specific B cells, are endocytosed and processed. Gliadin-DQ2/DQ8 complexes are then presented by the tTG-specific B cells to gliadin-specific T cells, a process that leads to the production of anti-tTG antibodies.

The presence of tTG-specific autoantibodies only in patients who have gluten in their diet suggests that the generation of such antibodies in CD requires gluten as an exogenous trigger. The proposed mechanism by which autoimmunity develops in CD is that the enzyme tTG generates additional antigenic epitopes by cross-linking gliadin peptides to itself and/or to other protein substrates, and this stimulates mucosal T cells to produce autoantibodies against tTG and gliadin [18] (Figure 1). Since the existence of tTG-specific T cells in the intestinal mucosa of untreated patients is not proven, it is hypothesized that the production of anti-tTG antibodies is driven completely by intestinal gliadin-specific T cells. The observation that anti-tTG antibody titers fall and can become undetectable during a gluten-free diet suggests that B cell activity depends on persistent antigen presentation. In a pioneering study in 1990, Porta et al. demonstrated that wheat glutelins and gliadins, as well as purified A-gliadin, act as acyl donor substrates for tTG [19]. In particular, by performing incubations *in vitro* both in the presence of radiolabeled polyamines and in their absence, Porta et al. showed that these proteins were able to produce not only *γ*(glutamyl)polyamine adducts but also polymeric complexes, probably through intermolecular *ε*(*γ*-glutamyl)lysine crosslinks. In the case of A-gliadin, the single lysil residue occurring in the amino acid sequence (K-186) is assumed to act as an acyl acceptor site. It is worth noting that the increase of both tTG activity *in situ* and tTG protein has been detected at critical sites of celiac mucosae, such as the intestinal brush border and subepithelial compartments [20]. 

The involvement of tTG in the pathogenesis of CD could be also due to another distinct but interdependent pathway via a gliadin-derived peptide deamidation reaction (Figure 1).Gluten peptides are specifically recognized by human leukocyte antigen (HLA)-DQ2/DQ8,a class II major histocompatibility complex [21]. Indeed, CD is strongly associated with the genes encoding HLA-DQ2, and gluten-specific CD4^+^ intestinal T cells can be isolated from intestinal biopsies of CD patients but not from healthy controls [21]. By contrast, there is no evidence of T cell-mediated reactivity against dietary gliadin in the nonceliac mucosa. Moreover, gliadin-specific T lymphocytes from CD intestinal mucosa are mainly of the Th_1_/Th_0_ phenotype, which after gliadin recognition, release prevalently proinflammatory cytokines dominated by interferon (IFN)-*γ* [22] and interleukin (IL)-10 [23]. It has been hypothesized that tTG might be responsible for the deamidation of specific glutamine residues within naturally digested gluten peptides, especially at low pH. Such tTG-catalyzed posttranslational modification generates negatively charged amino acid residues that bind with an increased affinity to the HLA-DQ2 or HLA-DQ8 molecules, thus potentiating T cell activation [24, 25]. Recognition of the T cell epitope has been particularly difficult since gliadin's peculiar amino acid composition and its high glutamine content make it an excellent tTG substrate. A 33 mer peptide, containing three of the most immunogenic epitopes, was identified as one of the main stimulators of the inflammatory response to gluten, resistant to intestinal proteases [26, 27]. However, Camarca et al. recently demonstrated that the repertoire of gluten peptides recognized by adult celiac patients is larger than had been previously thought, and it differs from one individual to another. Indeed, they found several active gluten peptides with a large heterogeneity of responses [28]. 

Although the role of gluten in activating gluten-specific T lymphocytes in the *lamina propria* is well established, it has been demonstrated that gluten contains peptides that can stimulate cells of the innate immune system. The prototype of innate peptides is peptide 31–43/49, which has been shown to be toxic for CD patients both *in vitro* and *in vivo* [29, 30]. Peptide 31–43/49 can reorganize intracellular actin filaments [31], induce maturation of bone-marrow-derived dendritic cells [32], and, by affecting epithelial growth factor-receptor decay, induce epithelial cell proliferation [33]. The peptide also stimulates the synthesis and release of the proinflammatory cytokine IL-15 that can promote an adaptive immune response [34] involving CD4^+^ T cells that recognize various deamidated gliadin peptides [25]. Most of the events related to innate immune activation were inhibited by antibodies neutralizing IL-15, thus confirming that this cytokine mediates intestinal mucosal damage induced by ingestion of gliadin. In particular, Barone et al. investigated the molecular mechanisms of the gliadin-induced IL15 increase and discovered that gliadin peptide 31–43 increases the levels of IL-15 on the cell surface of CaCo-2 cells probably by interfering with its intracellular trafficking [35].

## 4. Treatment of Celiac Disease

To date, no pharmacological treatment is available to gluten-intolerant patients. A strict, life-long, gluten-free diet is the only safe and efficient treatment available, although it results in a social burden. Adhering to a gluten-free diet can have a significant negative impact on perceived quality of life and may produce considerable psychological, emotional, and economic stress. Moreover, this requirement for dietary compliance is made more difficult by the exclusion of wheat, rye, and barley from the diet, which are important sources of iron, dietary fibre, and vitamin B, especially for adolescents and adults who need continuous monitoring by dieticians [36]. A lifelong gluten-free diet can be extremely difficult since gluten may be present in nonstarchy foods such as soy sauce and beer, as well as in nonfood items including some medications, postage stamp glue, and cosmetics (e.g., lipstick). CD patients can, therefore, be exposed inadvertently to gluten. Moreover, even after many years of gluten avoidance, CD patients never acquire tolerance to gliadin, and re-exposure to the antigen reactivates the disease. Finally, it is worth noting that a small group of patients with CD (2%–5%) fail to improve clinically and histologically upon elimination of dietary gluten. This complication is referred to as refractory CD, and it imposes a serious risk for developing lethal enteropathy-associated T-cell lymphoma. 

In 2000, the Codex Alimentarius Commission of the World Health Organization and the FAO described gluten-free foods consisting of, or made only from, ingredients which do not contain any prolamines from wheat or any Triticum species, such as spelt, kamut or durum wheat, rye, barley, oats, or their crossbred varieties with a gluten level not exceeding 20 ppm [37, 38]. At present, gluten-free products are not widely available; they are usually expensive, and they have poor sensory and shelf life properties. Research and development are currently focused on improving mouth-feel, flavour, and rheology of gluten-free products. Gluten is responsible for most of the viscoelastic properties of wheat flour doughs, and its absence can result in a baked bread with a crumbling texture, poor colour and other postbaking quality defects [37]. For all these reasons the search for safe and effective therapeutic alternatives to a gluten-free diet, which are compatible with a normal social lifestyle, is of great importance. Advances in our understanding of the complex mechanisms involved in CD pathogenesis have opened several promising avenues for therapeutic intervention aimed at targeting each factor involved in the disease onset, some of which are being tested in early clinical trials [39]. The identification of T-cell stimulatory gliadin sequences (33 mer peptide) is important so that peptide analogues of gliadin epitope(s) can be engineered to generate peptides that exert antagonistic effects. Of course, the chances of success of using peptide analogues to modulate specific immune responses could be hampered by the wide heterogeneity of the gliadin T-cell epitopes. Elucidation of the hierarchy of pathogenic gliadin epitopes and their core region is required before a peptide-based therapy can be designed [40]. Antibodies to IL-15 have also been proposed as a treatment strategy, particularly in cases of refractory sprue because of intraepithelial lymphocyte activation in this condition [41], as well as antibodies to IFN-*γ* [42]. Other promising treatment strategies are aimed at preventing gliadin presentation to T cells by blocking HLA-binding sites and using IL-10 as a tool for promoting tolerance [23]. To reverse the toxic effects induced by gliadin in human intestinal cells and gliadin-sensitive HCD4-DQ8 mice, Pinier et al. proposed a completely different strategy based on the use of synthetic sequestering polymeric binders that can complex and neutralize gliadin *in situ*. Coadministration of synthetic polymeric binders and gliadin to HLA-HCD4/DQ8 mice attenuated gliadin-induced changes in the intestinal barrier and reduced intraepithelial lymphocyte and macrophage cell counts [43]. Recent new therapeutic approaches include correction of the intestinal barrier defect against gluten entry. An intestinal permeability blocker (AT1001), which is an inhibitor of the zonulin pathway that acts to prevent gliadin from inducing increased intestinal permeability, is currently in a phase IIb clinical study [44]. Finally, a vaccine that could desensitize or induce tolerance in individuals with CD has been proposed [45, 46].

Besides therapeutic treatments, transgenic technology and breeding ancient varieties have been tried with the goal of developing grains that have a low or zero content of immunotoxic sequences, but with reasonable baking quality. However, these approaches are difficult due to the number and the repetition of sequence homologies in the cereal protein family, and because cereals like wheat are hexaploid [47, 48].

## 5. Enzyme Therapy

Enzyme supplement therapies are focused on inactivating immunogenic gluten epitopes (Table 1).

### 5.1. Oral Administration of Bacterial Endopeptidases

After ingestion, degraded gluten proteins reach the small intestine. However, because of their unusually high proline and glutamine content, especially in immunodominant gliadin peptides like the 33 mer, gluten is poorly degraded by the enzymes present in the gastrointestinal tract. Hence, oral enzyme therapy has been suggested as an alternative to the gluten-free diet. Promising enzymes (expressed in various microorganisms) tested are the prolyl oligopeptidases from *Flavobacterium meningosepticum*, *Sphingomonas capsulate*, and *Myxococcus xanthus*. These enzymes are capable of degrading proline-containing peptides that are otherwise resistant to degradation by proteases in the gastrointestinal tract *in vitro* [49–51]. However, most of these enzymes are irreversibly inactivated in the stomach by pepsin and acidic pH, thus failing to degrade gluten before it reaches the small intestine [49]. Encapsulation of these prolyl oligopeptidases was proposed in order to protect them from gastric juices [51]. However, in a recent *ex vivo* study, using biopsy-derived intestinal tissue mounted in Ussing chambers, it was observed that only high doses of prolyl oligopeptidase were capable of eliminating the accumulation of immunogenic peptides in the serosal compartment [50]. This indicates that, even if the enzyme were encapsulated, it is too inefficient to degrade gluten before it reaches the proximal part of the duodenum, the site where gluten triggers inflammatory T-cell responses [50]. Mitea et al. recently investigated a new prolyl endoprotease from *Aspergillus niger*. This enzyme was found to degrade gluten peptides and intact gluten proteins efficiently in the stomach, to such an extent that hardly any traces of gluten reached the duodenal compartment [52]. Moreover, the optimum pH of this enzyme is compatible with that found in the stomach and the enzyme is resistant to degradation by pepsin. Finally, prolyl endoprotease from *Aspergillus niger* is derived from the food grade microorganism and is available on an industrial scale. These results indicate that this enzyme might be suitable for oral supplementation to degrade gluten proteins in food before they reach the small intestine [52]. Recently, Gass et al. evaluated a new combination therapy, consisting of two gastrically active enzymes that detoxify gluten before its release in the small intestine. They used a glutamine-specific endoprotease (EP-B2; a cysteine endoprotease from germinating barley seeds) and a prolyl-specific endopeptidase from *Sphingomonas capsulata*, for its ability to digest gluten under gastric conditions. Endoprotease EP-B2 extensively hydrolyzes the gluten network in bread into relatively short (but still inflammatory) oligopeptides, whereas prolyl-specific endopeptidase from *Sphingomonas capsulata* rapidly detoxifies oligopeptides after primary proteolysis at internal proline residue level to yield nontoxic metabolites [53]. A practical advantage of this combination product is that both enzymes are active and stable in the stomach and can therefore be administered as lyophilized powders or simple capsules or tablets.

### 5.2. Pretreatment of Whole Gluten with Bacterial-Derived Peptidase

An alternative approach to detoxify gluten is represented by the digestion of wheat gluten peptides with bacterial-derived peptidase during food processing and before administration to patients. Traditional methods to prepare cereal foods, including long fermentation times by selected sourdough lactic acid bacteria, have mostly been substituted by the indiscriminate use of chemical and/or baker's yeast leavening agents. Under these circumstances, cereal components (e.g., proteins) are subjected to very mild or absent degradation during manufacture, resulting, probably, in reduced digestibility compared to traditional and ancient sourdough baked goods [54]. Di Cagno et al. selected four sourdough *lactobacilli* (*L. alimentarius *15M*, L. brevis *14G*, L. sanfranciscensis *7A, and* L. hilgardii* 51B) that showed considerable hydrolysis of albumin, globulin, and gliadin fractions during wheat sourdough fermentation. These *lactobacilli* had the capacity to hydrolyze the 31–43 fragment of A-gliadin *in vitro* and, after hydrolysis, greatly reduced the agglutination of K 562(S) subclone cells of human myelogenous leukemia origin by a toxic peptic-tryptic digest of gliadins [55]. On the basis of these results, and with the goal of decreasing gluten intolerance in humans, the authors investigated a novel bread making method that used selected *lactobacilli* to hydrolyze various Pro-rich peptides, including the 33 mer peptide, for the production of sourdoughs made from a mixture of wheat and nontoxic oat, buckwheat, and millet flours [56]. After 24 hours of fermentation, wheat gliadins and low-molecular-mass, alcohol-soluble polypeptides were almost completely hydrolyzed. Proteins extracted from sourdough were used for *in vitro* agglutination tests on K 562(S) subclone cells of human origin and to produce two types of bread, containing ca. 2 g of gluten. The latter were used in an *in vivo* double-blind acute challenge of CD patients. Agglutination testing of K 562(S) cells and the acute *in vivo* challenge showed improved tolerance of breads containing 30% wheat flour [56]. The reported data suggest that long-time fermentation in the presence of a mixture of selected lactic acid bacteria seemed to be indispensable to reduce toxicity. In actual fact, different probiotic bacterial strains have their characteristic set of peptidases, which may diverge considerably from each other and have variable substrate specificities. In line with this concept, different probiotic bacterial strains have been tested. Among probiotic preparations, VSL#3, a highly concentrated mixture of lactic acid and bifido-bacteria, was able to hydrolyze completely the *α*2-gliadin-derived epitopes 62–75 and 33 mer (750 ppm) [57]. It is interesting to underline that probiotics, defined as the viable microorganisms that exhibit a beneficial effect on the health of the host by improving its intestinal microbial balance, could directly modulate the function of epithelial cells. It has been reported that different probiotic strains, including the VSL3# preparation, increase epithelial barrier function by stabilizing tight junctions and inducing mucin secretion in epithelial cells [57, 58]. Furthermore, several probiotic bacterial strains are able to protect the epithelium, presumably by the aforementioned mechanisms, from various insults, including pathogenic bacteria [59, 60] and inflammatory cytokines [61, 62]. More recently, Rizzello et al. showed that fermentation with a complex formula of sourdough *lactobacilli* decreased the concentration of gluten to below 10 ppm [63]. Specifically, they used a mix of ten sourdough *lactobacilli* that were selected for their peptidase systems capability to hydrolyze Pro-rich peptides, including the 33 mer peptide, together with fungal proteases, that are routinely used as improvers in the baking industry. In this way, wheat and rye breads or pasta, if supplemented with gluten-free flour-based structuring agents, may be tolerated by CD patients. Agglutination testing of K 562(S) cells and an acute *in vivo* challenge showed improved tolerance of breads containing 30% wheat flour. Moreover, prolonged *in vivo* challenge of CD patients confirms reduced toxicity of gluten fermented with selected *lactobacilli* and fungal proteases. In fact, CD patients (age >12 years old) on a gluten-free diet for at least five years were challenged with a daily intake of 10 g of hydrolysed gluten (<20 ppm of gluten) for 2 months. Intestinal functional tests, as well as CD serum antibodies (anti-tTG, anti-endomysium), and duodenal histology and immunohistochemistry at the beginning and after 60 days of challenge showed that all parameters were normal, i.e., no villous atrophy) [64].

The use of proteases from germinating wheat seeds has also been proposed to create safe cereal products for CD patients [65, 66].

### 5.3. Transamidation of Gliadin

tTG-catalyzed deamidation of specific glutamine residues within naturally digested gluten peptides generates negatively charged amino acid residues that bind with an increased affinity to the HLA-DQ2/DQ8 molecules, thus potentiating T cell activation. Based on this assumption, Gianfrani et al. proposed an enzyme strategy to inactivate immunogenic peptide epitopes and, at the same time, to preserve the integrity of the protein structure *via* the transamidation of wheat flour with a food-grade enzyme and an appropriate amine donor [67]. Interestingly, a recent study showed that the formation of the DQ2-*α*-II epitope was blocked by 5-biotinamido pentylamine and by monodansylcadaverine, reagents known to cross-link glutamine residues [68]. To this end, the authors treated wheat flour with tTG and lysine methyl ester; the lysine-modified gliadin peptides lost almost completely their affinity to bind to HLA-DQ2. Moreover, lysine-modified gliadin peptides caused a drastic reduction in gliadin-specific IFN-*γ* production in intestinal T-cell lines derived from CD patients where the mucosal lesion was mainly induced by the production of IFN-*γ* from these gluten-specific T cells. This result suggests that transamidation neutralized the immune reactivity of a large repertoire of epitopes. Similar results were obtained by using microbial TG, which is different from tTG since it is calcium independent and is a low-molecular-weight protein that exhibits advantages in food industrial applications. This enzyme is commercially available as a dough improver that adds stability and elasticity to the dough. Additionally, bread volume and crumb texture are positively influenced by the addition of microbial TG, especially for flours with low gluten content and poor baking performance.

### 5.4. Transglutaminase Inhibitors

tTG plays an important role in CD pathology as it catalyzes deamidation and cross-linking of specific gluten peptides and converts them into potent epitopes recognized by intestinal T-cells. In order to restrain the T cell-mediated immune response to dietary gluten, a different approach could be to consider tTG as a potential therapeutic target [69]. The inhibition of the tTG-catalyzed deamidation of specific glutamine residues within naturally digested gluten peptides might not generate negatively charged amino acid residues and therefore might not increase the binding to the HLA-DQ2/DQ8 molecules (thus potentiating T cell activation). Several inhibitors acting with different mechanisms that target the TG cross-linking activity have been developed and tested, mainly *in vitro* [69, 70]. Among the tTG inhibitors tested we can find several nonspecific irreversible thiol-reactive reagents, also named suicide TG inhibitors, such as cystamine, able to inhibit tTG *via* the formation of an enzyme-inhibitor complex. Furthermore, we can find competitive nonpeptidic tTG amino donor substrates, such as 1,4-diaminobutane (Fibrostat), which is used topically in a clinical trial to treat abnormal wound healing [71], and competitive peptidic tTG amino donor. Finally, we can find amine acceptor pseudosubstrates able to inhibit tTG activity by diverting it from the natural protein substrate. Recently, Hoffmann et al. used a blocking peptide approach to reduce the processing of gliadin by tTG. The authors showed that these peptides have a potential for gluten detoxification and could be evaluated as an alternative for designing new food products for gluten-intolerant patients [72]. Several factors must be taken into consideration when designing a tTG inhibitor: inhibitory potency, optimal size of the compound, resistance towards intestinal proteolytic activities (to this end, amino acid residues will be replaced by peptidomimetics), and selectivity towards tTG. In fact, the lack of specificity limits therapeutic utility. Furthermore, to reduce the risk of systemic side effects, the activity of an optimal tTG inhibitor should be specifically limited to the compartment where gliadin encounters the immune system, that is, in the gut. Therefore, blocking the transamidating activity of tTG represents an attractive tool to prevent immune activation. Similar approaches have already been investigated in other diseases where tTG is involved, such as in the neurodegenerative disorders such as Parkinson, Huntington, and Alzheimer diseases, as well as in cancer and in fibrotic/scarring conditions such as diabetic nephropathy. Consequently, tTG inhibitory drugs can be predicted to have a wide medical application.

## 6. Concluding Remarks

The high incidence of CD in the worldwide population is a challenging task, given the negative impact of a strict gluten-free diet on the perceived quality of life of celiac patients for several reasons. A life-long gluten-free diet is not easy to maintain since gluten is the most common ingredient in the human diet. Multidisciplinary research efforts are currently being carried out in several directions to find new treatment strategies in order to reduce gluten toxicity. The use of oral proteases capable of detoxifying ingested gluten and new food grade fermentation technologies using bacterial-derived endopeptidases currently represent the most advanced and promising strategies.

## Figures and Tables

**Table 1:** Potential enzyme therapies for celiac disease.

Target for detoxification	Detoxifying agent	Mechanism of action	Status of research
Ingested gliadin peptides	Prolyl endopeptidases from:	Hydrolysis of proline-rich peptides of gliadin	Preclinical
	*S. capsulate* [49]		
	*F. meningosepticum *[50]		
	*M. xanthus *[51]		
	Prolyl endopeptidases from:* A. niger *[52]		Clinical trial
	Prolyl endopeptidases from: *S. capsulate *in combination with a glutamine-specific endoprotease (EP)-B2 from germinating barley [53]		Clinical trial

Flour	Sourdough lactobacilli-derived peptidases [56]	Hydrolysis of proline-rich peptides of gliadin	Clinical trial
	Sourdough lactobacilli-derived peptidases in combination with fungal proteases [64]		Clinical trial

Flour	Transglutaminase enzymes [67]	Transamidation of gliadin peptides with lysine methyl ester	Preclinical

Mucosal tTG	Irreversible thiol-reactive reagents, competitive peptidic, and nonpeptidic substrates [69]	unspecific or specific tTG inhibition	Preclinical

## Abbreviations

CD: Celiac disease
SS: Disulphide
tTG: Tissue transglutaminase
HLA: Human leukocyte antigen
IFN: Interferon
IL: Interleukin.
